# Analysis of the diagnostic value of peripheral blood immune inflammatory indicators of female bladder pain syndrome

**DOI:** 10.3389/fsurg.2025.1685098

**Published:** 2025-10-31

**Authors:** Yinglei Wang, Shenglai Liu, Haonan Shi, Chunxiao Xie, Peng Li, Kaipeng Jia, Yang Tang, Hailong Hu

**Affiliations:** 1 Pelvic Floor Dysfunction Diagnosis and Treatment Center, Department of Urology, The Second Hospital of Tianjin Medical University, Tianjin, China; 2 Pelvic Floor Dysfunction Diagnosis and Treatment Center, Department of Urology, Physical Examination Center, The Second Hospital of Tianjin Medical University, Tianjin, China

**Keywords:** female bladder pain syndrome (FBPS), interstitial cystitis/bladder painsyndrome (IC/BPS), blood inflammatory markers, maximum bladder capacity (MBC), neutrophil-to-lymphocyte ratio (NLR), systemic immune-inflammation index (SII)

## Abstract

**Introduction and hypothesis:**

Female bladder pain syndrome (FBPS), previously known as interstitial cystitis/bladder pain syndrome (IC/BPS). Numerous research indirectly prove that Female bladder pain syndrome (FBPS) is associated with immune-related inflammation. According to the correlation analysis between peripheral blood immune-inflammatory markers and disease diagnosis, this research further verifies the potential diagnostic value of peripheral blood inflammatory markers in FBPS.

**Method:**

A total of 149 women with bladder pain syndrome who visited the urology department of our hospital from January 2013 to December 2024 and 149 healthy controls Patients who underwent health examinations at the physical examination center of our hospital from January 2022 to January 2023 were screened. All patients' peripheral blood inflammatory markers at admission included Neutrophil-to-Lymphocyte ratio (NLR), Systemic Immune Inflammation index (SII), et al. The maximum bladder capacity (MBC) during surgery served as the bladder volume. Multivariate binary regression was used to calculate the correlation between these indicators and disease diagnosis as well as MBC. The correlation between these indicators and MBC is secondary outcomes. The optimal cut-off value for the parameters was identified using the receiver operating characteristic (ROC) curve and its area under the curve (AUC) over time.

**Results:**

Compared with the control group, patients in the observation group demonstrated significantly higher SII, NLR, PLR, neutrophil count, whereas peripheral blood platelet count (PLT) and absolute lymphocyte count decreased, with statistically significant differences (all *P* < 0.05). Multivariate binary logistic regression analysis revealed increased NLR as an independent risk factor for FBPS. Compared with the normal bladder capacity group, patients with small bladder capacity demonstrated significantly lower SII, PLR, PLT, with statistically significant differences (all *P* < 0.05). Multivariate binary logistic regression analysis revealed decreased PLT as an independent risk factor for reduced bladder capacity.

**Conclusion:**

Peripheral blood inflammation indicators can be employed as an auxiliary diagnostic standard for FPBS, and NLR can be used as an independent diagnostic indicator for FBPS. However, further prospective studies are warranted to identify the causal relationship of these indicators with patient symptoms.

## 1 Introduction

Female bladder pain syndrome (FBPS), previously known as interstitial cystitis/bladder pain syndrome (IC/BPS), is a life-altering and morbid condition that occurs primarily in female patients and can be variable in presentation. The International Urogynecological Association (IUGA) and the American Urogynecologic Society (AUGS) have updated the terminology of IC/BPS in order to improve the accuracy of diagnosis and the clinical care for affected female patients. Given the absence of pathognomonic symptoms and sensitive diagnostic tests, significant symptomatic overlap with numerous other pelvic conditions (such as pelvic floor tension myalgia or endometriosis) occurring in women makes diagnosis of FBPS challenging (1). Women are 2–5 times more likely to be affected than men (2). In the United States, 2%–6% of women demonstrated symptoms consistent with IC/BPS (3).

Research indicates that the neutrophil-to-lymphocyte ratio (NLR) holds significant predictive value in systemic inflammatory responses. An elevated NLR reflects increased neutrophil counts, suggesting that the body is in an inflammatory state. It has been demonstrated to have considerable predictive significance in cardiovascular and lower urinary tract diseases (4–8). Additionally, the platelet-to-lymphocyte ratio (PLR) reflects the body's coagulation status and inflammatory response. PLR has been confirmed as an independent risk factor for the occurrence and prognosis of diseases such as stroke (9, 10). The systemic immune-inflammation index (SII) is based on changes in immune cell subsets and platelet counts, serving as an indicator of overall platelet aggregation, activation, apoptosis, and alterations in the systemic immune microenvironment. An elevated SII suggests platelet activation due to systemic inflammation, accompanied by increased neutrophil and platelet counts. Conversely, a decreased SII indicates platelet aggregation or apoptosis, as well as changes in the systemic immune microenvironment caused by elevated lymphocyte counts. The systemic immune-inflammation index (SII) is a novel biomarker for malignancies and inflammatory diseases (11). These peripheral blood cell related indicators are relatively easy to obtain and more acceptable to patients as a non-invasive initial screening test.

FBPS symptoms overlap with those of other urological and gynecological disorders, including overactive bladder, recurrent urinary tract infections, vulvar pain, Endometriosis (12). Further, FBPS involves many comorbidities, including cardiovascular disease, lung disease, rheumatic diseases, neurological disorders, and mental health issues (13). FBPS is an exclusionary diagnosis due to the lack of identifiable symptoms and comorbidities, coupled with the absence of a gold standard diagnostic tool (14). Peripheral blood immune-inflammatory markers demonstrate diagnostic differences in various diseases, especially in immune-related diseases. Studies have confirmed that changes in the number of neutrophils and lymphocytes in the peripheral blood are associated with disease diagnosis despite no direct evidence associating FBPS with immune factors (15). Moreover, some articles have investigated the correlation between inflammatory and immune factors with FBPS pathogenesis. This paper primarily aims to verify the correlation between platelet count, neutrophil count, lymphocyte count, and their ratios with disease diagnosis, emphasizing the potential diagnostic value of these values.

## 2 Materials and methods

### 2.1 General information

We diagnosed with FBPS based on history taking, cystoscopy, and pathological examination following the diagnostic criteria for IC/BPS published by the American Urological Association (2011, 2022) (16, 17) and expert consensus developed by AUGS-IUGA (1). First, these patients associated with lower urinary tract symptoms (LUTS) for longer than 6 weeks, accompanied by at least one LUTS of urgency to void or frequency, in the absence of urinary tract infection (16). Collect the patient's medical history and admission examinations. Further, these patients met the National Institute of Diabetes and Digestive and Kidney Diseases (NIDDK) exclusion criteria at their first hospitalization (18).

Inclusion criteria

(1) age of >18 years; (2) first-time patient; (3) patients meeting the diagnostic criteria for FBPS; (4) patients with complete clinical data.

Exclusion criteria

(1) patients with acute or chronic bacterial cystitis, urinary stones, ketamine-related cystitis, or neurogenic urinary dysfunction and other urological diseases; (2) patients who have undergone urological surgery within the past 6 months; (3) patients who have received any therapeutic agent via cystoscopy within the past 6 months; (4) patients with concurrent inflammation of the female reproductive system or urinary tract tumors; (5) pregnant women and patients with allergic constitutions; (6) no increased nocturnal urination; (7) no more than 8 daytime urination when awake; (8) bladder or ureteral stones; (9) urethral diverticulum; (10) patients with immune system disease (e.g., Sjögren's syndrome, rheumatism diseases) and tuberculosis infection; (11) use of anti-inflammatory drugs (e.g., NSAIDs), immunosuppressants, or anticoagulants within 1–2 weeks prior to admission.

The blood routine and urine routine results of patients in the experimental group and control group were included in the statistics. Collect blood routine examination upon admission. Specific detection methods: Avoid the patient's menstrual period and collect 5 ml of morning venous blood, immediately performing routine blood tests. The white blood cell count (WBC), absolute lymphocyte count, absolute neutrophil count, PLT were measured using a fully automated biochemical analyzer. Routine urinalysis excludes urinary tract infection.

Collect the surgical records and postoperative pathological reports of these patients. Cystoscopy with one or more inflammatory appearing lesions or ulcerations (Hunner's lesion) was applied as the diagnostic criteria (16). Glomerulations (pinpoint petechial hemorrhages) after bladder hydrodistension were considered as another diagnostic criteria (Supplementary Figures 1A–C). All operators must adhere to the following standards and procedures: The bladder was then distended with normal saline through a port on the cystoscope at a pressure of 80 cm H2O. After hydrodistension for 3 min at the maximum bladder capacity, the saline was drained, and the volume of this fluid served as the intraoperatively maximum bladder capacity (MBC) (14, 19). We used biopsy forceps to remove urothelial tissue at inflammatory appearing lesions or ulcerations (Hunner's lesion), taking 2–4 pieces ranging from 2 mm to 3 mm. At least one pathological diagnosis in all the tissue met the following clinical pathological standards: multiple inflammatory cell infiltration in the mucosa and submucosa, including neutrophils, lymphocytes, and mast cells. Immunohistochemistry confirmed positive CD38, CD117, CD138, and LCA antibodies. A pathologist reviewed the pathology section and report for a second time.

Collect medical records of 200 patients, 23 patients with acute or chronic bacterial cystitis, urinary calculi, ketamine-associated cystitis, neurogenic urinary dysfunction, and other urological diseases; 9 patients with concurrent female reproductive system inflammation or urinary tract tumors; 14 patients with immune system disease (e.g., Sjögren's syndrome, rheumatism diseases). There are four of them used anti-inflammatory drugs or immunosuppressants, within 1–2 weeks prior to hospitalization. The remaining criteria did not exclude patients. No inflammatory appearing lesions or ulcerations (Hunner's lesion)were observed in 5 patients under cystoscopy. The observation group included 149 female patients who visited the urology department of our hospital from January 2013 to December 2024. Participants were aged 33–80 years [median 63 years; mean 61 years].

Self-reported questionnaires, including O'Leary–Sant's symptom and problem indexes (OSSI and OSPI), pelvic pain and urgency/frequency (PUF) symptom scale, documented the presence of FBPS indicative symptoms in the control groups, including dyspareunia, vaginitis, pelvic pain, frequent urination, urgency, nocturia (17, 20). The questionnaire scores of the control patients were OSSI + OSPI < 3 points, PUF < 3points. The control group included 149 female participants who underwent health examinations at the physical examination center of our hospital from January 2022 to January 2023. Participants were aged 35–84 years [median 60 years; mean 60 years].

To clarify the correlation between peripheral blood indicators and bladder capacity in experimental patients. Since bladder capacity was not statistically measured in the early cystoscopy, this data was indeed available for some of the experimental group patients. Finally, the MBC of 119 patients was statistically analyzed, with its median (320 ml, 200 ml–500 ml) used as the threshold. Patients with a level greater than the threshold were defined as having normal bladder capacity (NBC), whereas those with a value less than the threshold were defined as having small bladder capacity (SBC). The ratio of these two groups was 58:61.

General clinical data of patients and healthy people were collected, including age, body mass index (BMI), menopause, medical history, and routine urine and blood results. Clinical characteristics of the human subjects are presented in Supplementary Appendices Table 1.

### 2.2 Statistical methods

The Statistical Package for the Social Sciences version 24.0 statistical software was used for all data analyses. The Shapiro–Wilk test was used to identify whether the data followed a normal distribution. Normally distributed data were expressed as mean ± standard deviation, whereas nonnormally distributed data were presented as median (interquartile range). Mann–Whitney rank-sum tests or Kolmogorov–Smirnov tests were selected if two sets of data did not conform to a normal distribution.

Parametric *t*-tests were performed if both sets of data followed a normal distribution. Subsequent homogeneity of variance tests were conducted. Nonpaired *t*-tests were selected if the variances were homogeneous, whereas Welch's adjusted nonpaired *t*-tests were chosen if the variances were heterogeneous. A logistic regression model was developed to analyze the risk factors for FBPS in women. Receiver operating characteristic (ROC) curves were used to assess the diagnostic performance of peripheral blood indicators. *P*-values of <0.05 indicated statistical significance.

## 3 Results

### 3.1 Female bladder pain syndrome vs. healthy population

Comparison of clinical data and laboratory indicators between the two groups revealed no statistically significant differences in terms of age, BMI, history of hypertension and diabetes, and menopause compared with the control group (all *P* > 0.05), indicating comparability. The observation group demonstrated increased SII, NLR, PLR, absolute neutrophil counts, and significantly decreased platelet count and absolute lymphocyte count, with statistically significant differences (all *P* < 0.05) (Table 1, Supplementary Tables 2, 3). Logistic regression model using FBPS as the dependent variable and SII, NLR, and PLR for multivariate binary logistic regression analysis.

**Table 1:** Comparison of clinical data and laboratory indexes between the two groups.

		BMl/(kg/m^2^)	SII	NLR	PLR
FBPS	149	23.81 ± 3.20	530.71 ± 269.32	2.25 ± 0.88	153.81 ± 63.44
C	149	24.28 ± 3.79	399.34 ± 166.56	1.57 ± 0.48	123.79 ± 37.76
*P*		=0.416	<0.001	<0.001	<0.001
		N/(10^9^/L)	L/(10^9^/L)	PLT/(10^9^/L)	
FBPS		3.47 ± 1.03	1.66 ± 0.53	232.57 ± 55.29	
C		3.22 ± 0.97	2.11 ± 0.49	250.10 ± 56.54	
*P*		<0.05	<0.001	<0.05	

FBPS, female bladder pain syndrome Patients; C, controls patients; BMI, body mass index; S**II**, systemic immune inflammation index; NLR, neutrophil-to-lymphocyte ratio; PLR, platelet -to-lymphocyte ratio; N, neutrophil count; L, absolute lymphocyte count; PLT, peripheral blood platelet count.

**Figure 1:**
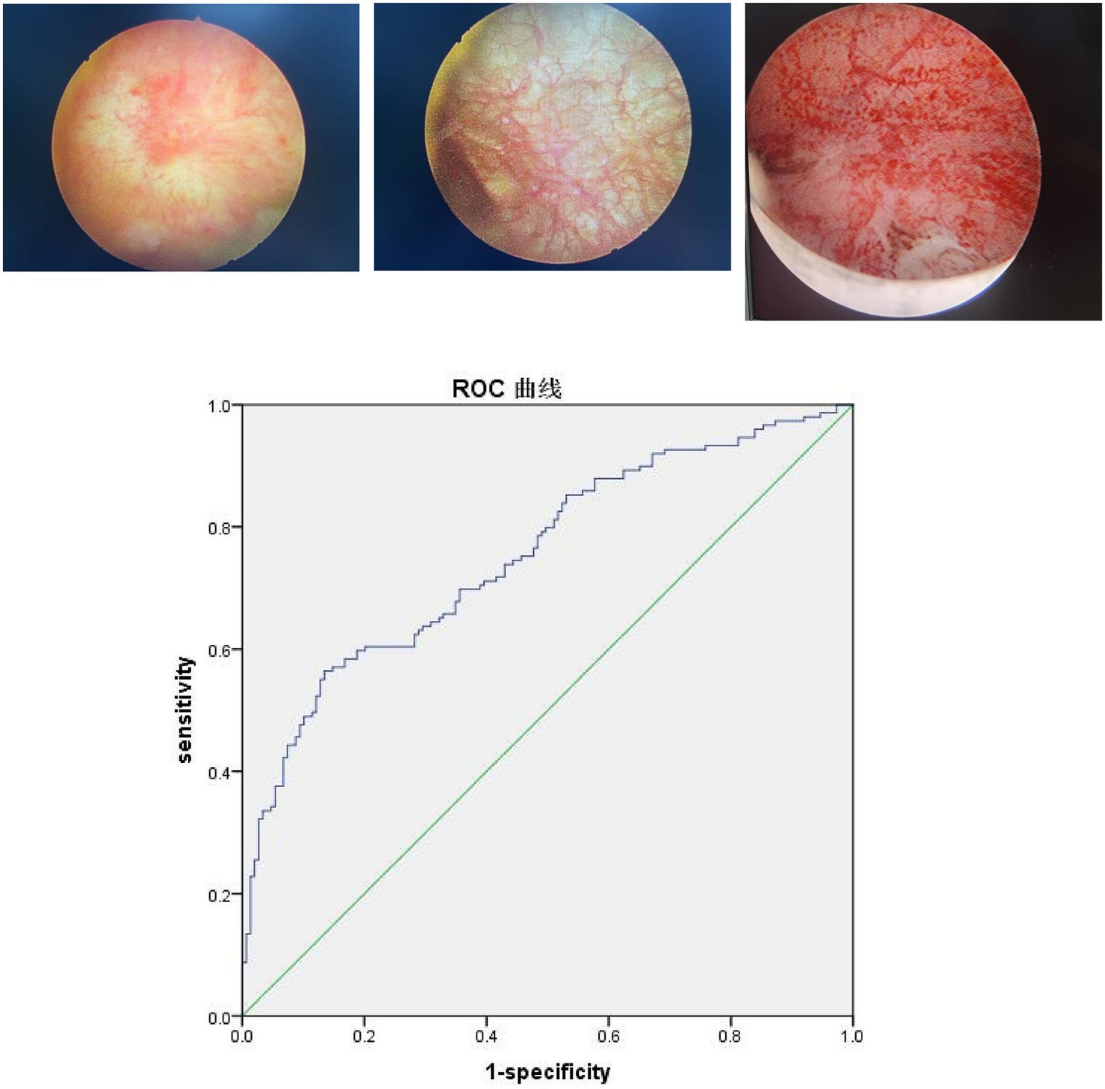
ROC curve analysis of plasma NLR. Three cystoscopy images show a Hunner's ulcer, non-ulcerated mucosa with reticulated, thickened blood vessels, and extensive bleeding after bladder hydrodistension. Below these is a Receiver Operating Characteristic (ROC) curve plotting sensitivity against 1-specificity. The blue curve lies above the green diagonal reference line, indicating good predictive performance. The diagonal line represents random probability.

The results reveal that an increased NLR level is an independent risk factor for FBPS. The ROC analysis indicates that the area under the curve (AUC) for diagnosing FBPS using NLR is 0.754 [95% confidence interval (CI): 0.700–0.809], which has a diagnostic value. When the optimal cut-off value is set at 2.065, the sensitivity is 0.557, the specificity is 0.866, and Youden's index is 0.423 (Table 2, Figure 1). The ROC analysis of the combined indicator NLR and PLT that the area under the curve (AUC) for diagnosing FBPS using is 0.554.

**Table 2:** Multivariate logistic regression analysis of plasma SII and other clinical parameters affecting FBPS.

	*B*	Std. error	Wald	*Df*	*P*	OR	HR (95%)	
							LL	UL
SII	−0.007	.002	20.634	1	.000	.993	.989	.996
NLR	3.340	.520	41.165	1	.000	28.206	10.169	78.232
PLR	.010	.004	5.459	1	.019	1.010	1.002	1.019

SII, systemic immune inflammation index; NLR, neutrophil-to-lymphocyte ratio; PLR, platelet -to-lymphocyte ratio.

### 3.2 Normal bladder capacity vs. small bladder capacity

No statistically significant differences in terms of age, height, weight, BMI, history of hypertension and diabetes, and menopausal status were found between the two groups (all *P* > 0.05), indicating comparability. Statistical analysis was conducted on the data from both groups. The neutrophil count, SII, PLR and PLT were decreased in patients with reduced bladder capacity, and the difference was statistically significant (*P* < 0.05) (Table 3, Supplementary Tables 4, 5).

**Table 3:** Comparison of normal bladder capacity and bladder capacity reduction in patients.

	BMI/(kg/m^2^)		SII	PLR	NLR
NBC	58	23.73 ± 2.65	606.03 ± 308.86	173.91 ± 69.44	2.35 ± 0.94
SBC	61	23.40 ± 3.89	442.08 ± 213.90	131.99 ± 40.89	2.09 ± 0.84
*P*		0.471	<0.05	<0.001	0.167
L{[*M*(*P*25, *P*75)]10^9^/L}		N{[*M*(*P*25, *P*75)]10^9^/L}		PLT{[*M*(*P*25, *P*75)]10^9^/L}	
NMBC	1.57 (1.17, 1.98)	3.36 (2.85, 4.14)		248.50 (215.00, 283.50)	
SMBC	1.61 (1.35, 2.08)	3.25 (2.36, 4.09)		211.00 (176, 235.50)	
*P*	0.736	0.455		<0.001	

NBC, normal bladder capacity; SBC, small bladder capacity; MI, body mass index; SII, systemic immune inflammation index; NLR, neutrophil-to-lymphocyte ratio; PLR, platelet -to-lymphocyte ratio; N, neutrophil count; L, absolute lymphocyte count; PLT, peripheral blood platelet count.

**Figure 2:**
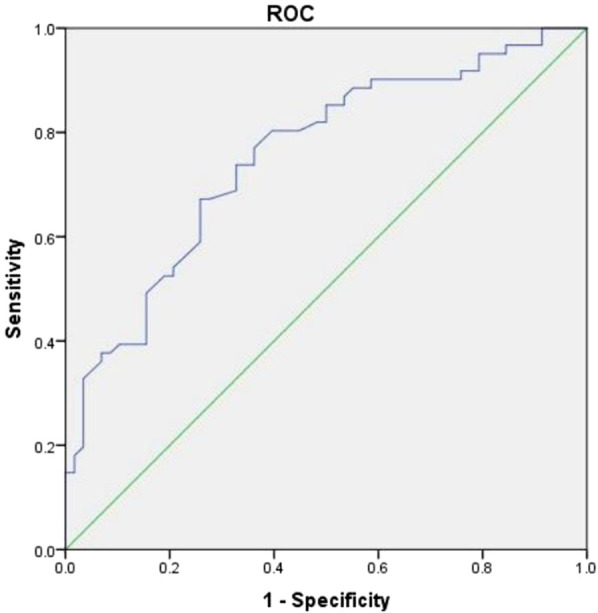
ROC curve analysis of plasma count for reduced bladder capacity deagnosis. The Receiver Operating Characteristic (ROC) curve plots sensitivity against 1-specificity. The curve is convex toward the upper-left corner, indicating excellent model performance.

The logistic regression model used MBC as the dependent variable and SII, PLR and PLT for multivariate binary logistic regression analysis. The results revealed a decrease in the platelet count as an independent risk factor for patients with FBPS. The ROC curve analysis indicated that the AUC for diagnosing bladder capacity reduction using platelet count was 0.754 (95% CI: 0.668–0.841), indicating a certain diagnostic value. Sensitivity, specificity, and Youden's index were 0.672, 0.741, and 0.413, respectively, when the optimal cut-off value was set at 220.5 (10^9^/L) (Table 4, Figure 2).

**Table 4:** Multivariate logistic regression analysis of plasma SII and other clinical parameters affecting small bladder capacity.

	*B*	Std. error	Wald	*Df*	*P*	OR	HR (95%)	
							LL	UL
SBCSII	.002	.001	1.292	1	.256	1.002	.999	1.004
SBCPLT	−.018	.006	10.838	1	.001	.982	.971	.993
SBCPLR	−.012	.006	4.055	1	.044	.988	.977	1.000

SII, systemic immune inflammation index; NLR, neutrophil-to-lymphocyte ratio; PLR, platelet -to-lymphocyte ratio.

The above results mean that combined with the questionnaire survey, if the peripheral blood routine test of the patient indicates NLR > 2.065, it can be highly suspected that the patient is FBPS. On this basis, if PTL < 220.5 (10^9^/L), then the bladder capacity of the patient is reduced, which is the cause of aggravating the symptoms of the patient, such as urinary frequency and urgency, etc.

## 4 Discussion

Concerning the etiology and pathogenesis of FBPS, various hypotheses have been proposed. Numerous studies investigated the theories of epithelial permeability, mast cell activation, and immune-neuro inflammation to explain various symptoms in patients, such as urinary frequency, urgency, dysuria, and bladder contracture, from different perspectives (21). There is controversy over the disease onset; however, chronic Inflammation and the immune system may play an indispensable role in its progression (22). Increased levels of various proinflammatory cytokines have been observed in the urine and serum of patients (23). Immune cell activity and structural abnormalities have been noted in tissue samples from patients (24). Some studies have revealed that protein factors, such as AFP and PPARγ, can serve as diagnostic markers for FBPS, providing significant advantages in sensitivity and specificity, but the testing process is complex and time-consuming (15, 24). A simple and rapid indicator to enable early FBPS diagnosis is now an urgent need.

Peripheral blood cell counts and ratios represent the long-term inflammatory cell status in patients (25). Chronic bladder inflammation promotes the activation and aggregation of neutrophils, lymphocytes, and eosinophils in the mucosa. The dilation of blood vessels in the bladder wall accelerates the extravasation of inflammatory factors, which in turn promotes the proliferation of inflammatory cells in the blood, particularly neutrophils. The pathological tissue report showed “vascular dilation with inflammatory cell infiltration” under the microscope, indicating that local inflammatory cells migrated to peripheral blood vessels and lymphatic vessels through bladder mucosal blood vessels and lymphatic vessels, causing changes in inflammatory cells and inflammatory factors in peripheral blood. The proliferation of inflammatory cells, especially neutrophils, in the blood leads to the elevation of NLR (26). The pathology indicates widespread inflammatory cell aggregation at the lesion site, however, this study shows a significant decrease in systemic lymphocyte levels, which may be associated with lymphocyte aggregation near the lesion. Further, prolonged inflammation increases oxidative stress in the blood, causing excessive ROS production that damages platelet survival and accelerates platelet aging and rupture.

This study revealed significantly reduced platelet count in the disease group, with a more pronounced decline observed in patients with bladder contracture. Systemic thrombocytopenia increases the risk of postoperative bleeding, which is a key consideration when assessing the feasibility of lesion resection and hydrodistension therapy. In patients with bladder contracture, a significant reduction in peripheral blood neutrophils is also observed. This suggests either systemic inflammatory cell recruitment to the lesion site or deterioration of the bladder mucosal immune microenvironment during advanced disease stages, correlating with aggravated local symptoms such as urinary frequency and dysuria. In later stages, increased urinary frequency may also correlate with reduced bladder capacity. Follow-up results indicate that while some patients experience transient symptom relief after bladder hydrodistension, others show minimal or no improvement. This necessitates further follow-up to clarify the relationship between patients' peripheral blood cell counts and the efficacy of hydrodistention. This allows for better selection of the optimal timing for hydrodistension.

This study has certain limitations. First, the research primarily focuses on female patients, thus, the sample limitations mean the findings can only be reflective of the underlying factors in this specific cohort. Future studies should include male patients with FBPS to verify the generalizability of the results. Second, the retrospective design and single-center sample limit the generalizability of this study and the control group in this study only included healthy individuals and did not include patients with other urological diseases. This design limitation means the specificity of the currently determined cut-off value for distinguishing FBPS from other inflammatory urological diseases like OAB remains unclear. This is important for the differential diagnosis of lower urinary tract diseases. Future prospective, multicenter studies with broader populations are required to validate these findings. Third, If possible, the disease classification and staging of the patient population in this study can be divided. This provides a good support for the choice of follow-up treatment plan for patients with disease. So the subsequent research should aim to expand the sample size and attempt to classify the patient population using survey questionnaires and the ESSIC guidelines. Additionally, integrating multi-omics data (e.g., urinary and pathological specimens) is essential to further investigate the diagnostic performance of combined inflammatory biomarkers and elucidate underlying disease mechanisms. In this article, NLR predicts disease specificity is high but sensitivity is low, until more precise peripheral blood indicators emerge, clinical diagnosis should be combined with questionnaire survey to avoid missed diagnosis.

The innovation of this paper lies in the first application of peripheral blood indicators from FBPS for differential exploration, thereby clarifying the trends of inflammatory cell proliferation and reduction in patients. This lays the foundation for further validation of the association between inflammation and symptoms. Moreover, we further explored the association between changes in inflammatory cells and bladder capacity alterations. This highlights that reduced bladder capacity in advanced-stage disease serves as a contributing factor to patients' discomfort symptoms, thereby informing the selection of clinical management options. Furthermore, this study explores the therapeutic potential of platelet-targeted local and systemic interventions in FBPS management. Platelet-rich plasma (PRP) contains many growth factors (platelet-derived growth factor PDGF) and cytokines that are essential proteins for modulating inflammation and promoting tissue regeneration and thus wound healing. PDGF is one of the several growth factors that regulate cell growth and division. There have been articles about the therapeutic effect of intravesical injection of enriched platelet plasma on FBPS (27, 28). By augmenting platelet availability in both circulatory and bladder microenvironments, these strategies aim to counteract the progressively deteriorating immune milieu, offering more effective treatment paradigms for patients. Although PLT has moderate sensitivity and specificity for predicting bladder capacity, but it was possible to explain the need for cystoscopy to patients at the initial screening, and to add bladder hydrodistension and platelet-rich plasma (PRP) treatment during cystoscopy.

## 5 Conclusion

This study provides new information about the pathophysiological mechanism of FBPS and preliminarily establishes its potential diagnostic markers. The NLR can provide an hematological adjunct to diagnosis when we strongly suspect the patient of FBPS. The prediction of bladder capacity by PLT, as a secondary conclusion, can better explain the patient's symptoms. This finding provides a new direction for FBPS diagnosis and treatment in the future, but more in-depth research is warranted for verification.

## Data Availability

The original contributions presented in the study are included in the article/Supplementary Material, further inquiries can be directed to the corresponding authors.

## Supplementary material

The Supplementary Materialfor this article can be found online at: https://www.frontiersin.org/articles/10.3389/fsurg.2025.1685098/full#supplementary-material

## References

[1] AUGS-IUGA Joint Publication. Joint terminology report: terminology standardization for female bladder pain syndrome. Int Urogynecol J. (2025) 36:265–77. 10.1007/s00192-024-05923-z 39751633

[2] Malde S Palmisani S Al-Kaisy A Sahai A. Guideline of guidelines: bladder pain syndrome. BJU Int. (2018) 122(5):729–43. 10.1111/bju.14399 29777618

[3] Suskind AM Berry SH Ewing BA Elliott MN Suttorp MJ Clemens JQ. The prevalence and overlap of female bladder pain syndrome and chronic prostatitis/chronic pelvic pain syndrome in men: results of the RAND interstitial cystitis epidemiology male study. J Urol. (2013) 189(1):141–5. 10.1016/j.juro.2012.08.088 23164386 PMC3894747

[4] Machado GP de Araujo GN Carpes CK Lech M Mariani S Valle FH Comparison of neutrophil-to-lymphocyte ratio and mean platelet volume in the prediction of adverse events after primary percutaneous coronary intervention in patients with ST-elevation myocardial infarction. Atherosclerosis. (2018) 274:212–7. 10.1016/j.atherosclerosis.2018.05.022 29803159

[5] Pinheiro Machado G Araujo GN Carpes CK Lech MC Mariani S Valle FH Elevated neutrophil-to-lymphocyte ratio can predict procedural adverse events in patients with ST-elevation myocardial infarction undergoing primary percutaneous coronary intervention. Coron Artery Dis. (2019) 30(1):20–5. 10.1097/mca.0000000000000671 30334819

[6] Liu W Wang J Wang M Ding X Wang M Liu M. Association between immune-inflammatory indexes and lower urinary tract symptoms: an analysis of cross-sectional data from the US National Health and Nutrition Examination Survey (2005–2008). BMJ Open. (2024) 14(3):e080826. 10.1136/bmjopen-2023-080826 38521530 PMC10961552

[7] Thorup CV Christensen S Hvas AM. Immature platelets as a predictor of disease severity and mortality in sepsis and septic shock: a systematic review. Semin Thromb Hemost. (2020) 46(3):320–7. 10.1055/s-0039-3400256 31858518

[8] Wu C-C Wu C-H Lee C-H Cheng C-I. Association between neutrophil percentage-to-albumin ratio (NPAR), neutrophil-to-lymphocyte ratio (NLR), platelet-to-lymphocyte ratio (PLR) and long-term mortality in community-dwelling adults with heart failure: evidence from US NHANES 2005–2016. BMC Cardiovasc Disord. (2023) 23(1):312. 10.1186/s12872-023-03316-6 37344786 PMC10286403

[9] Hu B Yang X-R Xu Y Sun Y-F Sun C Guo W Systemic immune-inflammation index predicts prognosis of patients after curative resection for hepatocellular carcinoma. Clin Cancer Res. (2014) 20(23):6212–22. 10.1158/1078-0432.Ccr-14-0442 25271081

[10] Ma R Cui L Cai J Yang N Wang Y Chen Q Association between systemic immune inflammation index, systemic inflammation response index and adult psoriasis: evidence from NHANES. Front Immunol. (2024) 15:1323174. 10.3389/fimmu.2024.1323174 38415255 PMC10896999

[11] Dziedzic EA Gąsior JS Tuzimek A Paleczny J Junka A Dąbrowski M Investigation of the associations of novel inflammatory biomarkers-systemic inflammatory Index (SII) and systemic inflammatory response index (SIRI)-with the severity of coronary artery disease and acute coronary syndrome occurrence. Int J Mol Sci. (2022) 23(17):9553. 10.3390/ijms23179553 36076952 PMC9455822

[12] Collings R Healey M Dior U Erwin R Rosamilia A Cheng C. Effect of investigative laparoscopy on bladder pain syndrome: a prospective cohort trial. Int Urogynecol J. (2020) 31(8):1583–91. 10.1007/s00192-019-04023-7 31240363

[13] Warren JW Howard FM Cross RK Good JL Weissman MM Wesselmann U Antecedent non bladder syndromes in case-controlstudy of interstitial cystitis/painful bladder syndrome. Urology. (2009) 73(1):52–7. 10.1016/j.urology.2008.06.031 18995888

[14] Guo Y Na Y Ye Z Huang J Zhang X. Guidelines for diagnosis and treatment of urology and andrology diseases in China (2022).

[15] Wang Z Zuo JP Che H Reng LY Xu Z Wang L. Evaluation of plasma PPARγ as a potential diagnostic marker for interstitial cystitis/cystodynia syndrome in women and its predictive value. J Pract Med. (2025) 41(2):258–63. Available online at: https://link.cnki.net/urlid/44.1193.r.20250107.1020.006

[16] Hanno PM Burks DA Clemens JQ Dmochowski RR Erickson D FitzGerald MP AUA guideline for the diagnosis and treatment of interstitial cystitis/bladder pain syndrome. J Urol. (2011 Jun) 185(6):2162–70. 10.1016/j.juro.2011.03.064 21497847 PMC9341322

[17] Clemens JQ Erickson DR Varela NP Lai HH. Diagnosis and treatment of interstitial cystitis/bladder pain syndrome. J Urol. (2022) 208(1):34–42. 10.1097/JU.0000000000002756 35536143

[18] Gillenwater JY Wein AJ. Summary of thenational institute of arthritis, diabetes, digestive andkidney diseases workshop on interstitial cystitis, National Institutes of Health, Bethesda, Maryland, August 28–29, 1987. J Urol. (1988) 140:203–6. 10.1016/S0022-5347(17)41529-1 3379688

[19] Niimi A Nomiya A Yamada Y Suzuki M Fujimura T Fukuhara H Hydrodistension with or without fulguration of hunner lesions for interstitial cystitis: long-term outcomes and prognostic predictors. Neurourol Urodyn. (2016) 35(8):965–9. 10.1002/nau.22837 26208131

[20] Parsons CL Dell J Stanford EJ Bullen M Kahn BS Waxell T Increased prevalence of interstitial cystitis: previously unrecognized urologic and gynecologic cases identified using a new symptom questionnaire and intravesical potassium sensitivity. Urology. (2002) 60(4):573–8. 10.1016/s0090-4295(02)01829-0 12385909

[21] Nambiar AK Arlandis S Bø K Cobussen-Boekhorst H Elisabetta Costantini E de Heide M European association of urology guidelines on the diagnosis and management of female non-neurogenic lower urinary tract symptoms. Part 1: Diagnostics, overactive bladder, stress urinary incontinence, and mixed urinary incontinence. Eur Urol. (2022) 82(1):49–59. 10.1016/j.eururo.2022.01.045 35216856

[22] Diniz BS Butters MA Albert SM Dew MA Reynolds CF III. Late-life depression and risk of vascular dementia and Alzheimer’s disease: systematic review and meta-analysis of community-based cohort studies. Br J Psychiatry. (2013) 202(5):329–35. 10.1192/bjp.bp.112.118307 23637108 PMC3640214

[23] Vera PL Preston DM Moldwin RM Erickson DR Mowlazadeh B Ma F Elevated urine levels of macrophage migration inhibitoryfactor in inflammatory bladder conditions: a potentialbiomarker for a subgroup of interstitial cystitis/bladderpain syndrome patients. Urology. (2018) 116:55–62. 10.1016/j.urology.2018.02.039 29580781 PMC5975106

[24] Maeda D Akiyama Y Morikawa T Kunita A Ota Y Katoh H Hunner-type (classic) interstitial cystitis: a distinct inflammatory disorder characterized by pancystitis, with frequent expansion of clonal B-cells and epithelialdenudation.PLoS One. (2015) 10(11):e0143316. 10.1371/journal.pone.0143316 26587589 PMC4654580

[25] Keay SK Zhang C-O Shoenfelt J Erickson DR Whitmore K Warren JW Sensitivity and specificity of antiproliferative factor, heparin-binding epidermal growth factor-like growth factor, and epidermal growth factor as urine markers forinterstitial cystitis. Urology. (2001) 57(6Suppl1):9–14. 10.1016/S0090-4295(01)01127-X 11378043

[26] Liu ZY Liu DG. Clinicopathological observation of 33 cases of interstitial cystitis. J Clin Exp Pathol. (2024) 40(4):369–73. 10.13315/j.cnki.cjcep.2024.04.007

[27] Kuo HC. Intravesical injections of autologous platelet-rich plasma for the treatment of refractory interstitial cystitis. Low Urin Tract Symptoms. (2023) 15(6):210–5. 10.1111/luts.12504 37702275

[28] Jiang Y-H Kuo Y-C Jhang J-F Lee C-L Hsu Y-H Ho H-C Repeated intravesical injections of platelet-rich plasma improve symptoms and alter urinary functional proteins in patients with refractory interstitial cystitis. Sci Rep. (2020) 10(1):15218. 10.1038/s41598-020-72292-0 32939046 PMC7495440

