# Supplementary material for: Analysis of the diagnostic value of peripheral blood immune inflammatory indicators of female bladder pain syndrome
Source: Front Surg. 2025 Oct 31;12:1685098. doi: 10.3389/fsurg.2025.1685098 (PMC12615403; doi:10.3389/fsurg.2025.1685098)
Supplement: Supplementary file 1 [file Supplementaryfile1.zip › Appendices Table/Appendices Table1.pdf]

Eq.1

	Quantity	Menopause	Hypertension	Diabetes Mellitus
FBPS	149	26	37	30
C	149	26	33	27

FBPS Female bladder pain syndrome Patients ;C controls Patients

Appendices. B

	Quantity	Menopause	Hypertension	Diabetes Mellitus
NBC	58	9	16	12
SBC	61	8	15	10

Appendices. C

Tests of Normality

	Kolmogorov-Smirnov			Shapiro-Wilk		
	Statistic	df	Sig.	Statistic	df	Sig.
ICY	.099	149	.001	.948	149	.000
ICBMI	.075	149	.039	.958	149	.000
ICSII	.096	149	.002	.872	149	.000
ICN	.053	149	.200*	.979	149	.020
ICL	.096	149	.002	.929	149	.000
ICPLT	.088	149	.007	.977	149	.012
ICNLR	.079	149	.025	.924	149	.000
ICPLR	.093	149	.003	.931	149	.000
CY	.064	149	.200*	.977	149	.013
CBMI	.069	149	.078	.976	149	.011
CSII	.072	149	.056	.954	149	.000
CN	.077	149	.032	.974	149	.006
CL	.141	149	.000	.916	149	.000
CPLT	.057	149	.200*	.948	149	.000
CNLR	.105	149	.000	.974	149	.006
CPLR	.046	149	.200*	.965	149	.001

$P<0.05$

Appendices. D

DESCRIPTIVES

	Quantity	Mean	SE.	Min.	Max.	Percentile		
						25%	50%	75%
ICY	149	60.47	9.721	31	80	56.00	62.00	67.00
ICBMI	149	23.8179	3.20420	15.15	38.63	21.6950	23.4400	26.0400
ICSII	149	506.3064	270.39797	105.73	2079.31	307.6247	452.3244	636.7839
ICN	149	3.3857	1.07351	1.34	6.60	2.5100	3.3000	4.0150
ICL	149	1.7315	.62181	.57	4.48	1.3000	1.6100	2.0800
ICPLT	149	232.8591	55.55506	113.00	400.00	188.5000	222.0000	270.0000
ICNLR	149	2.1432	.90326	.54	5.32	1.4962	2.0724	2.6115
ICPLR	149	149.8330	60.65494	59.54	389.47	106.5997	138.1503	179.8020
CY	149	59.50	10.246	37	88	53.50	60.00	66.00
CBMI	149	24.2809	3.79090	17.60	34.66	21.3323	23.9420	26.5136
CSII	149	399.3430	166.55656	125.38	980.28	269.3634	379.6652	491.0333
CN	149	3.2185	.96538	1.48	7.95	2.4850	3.2000	3.7750
CL	149	2.1132	.49728	1.01	3.49	1.7800	2.0000	2.4650
CPLT	149	250.1007	56.54091	131.00	489.00	210.0000	245.0000	284.0000
CNLR	149	1.5717	.48049	.54	3.37	1.2308	1.5122	1.9002
CPLR	149	123.7917	37.76252	59.82	285.15	98.9609	116.8421	141.3769

Appendices. E

	Quantity	Mean	SE	Min	Max	Percentile 25%	50%	75%
NBCY	58	60.88	10.762	32	77	55.75	63.00	68.00
NBCBMI	58	23.7807	2.63217	19.10	33.73	21.9075	23.4300	25.5450
NBCSII	58	597.9081	308.31800	195.13	2079.31	406.3827	553.6409	699.8767
NBCN	58	3.6433	1.07452	1.34	6.40	2.8800	3.6500	4.2200
NBCL	58	1.7048	.57921	.89	3.61	1.2350	1.6050	1.9825
NBCPLT	58	254.8621	48.96775	167.00	400.00	219.5000	250.0000	283.5000
NBCNLR	58	2.3075	.90747	.79	5.32	1.7430	2.1342	2.6665
NBCPLR	58	166.1859	63.12841	61.56	344.83	118.2069	156.9984	208.6757
SBCY	61	61.30	8.909	31	80	57.00	63.00	67.00
SBCBMI	61	23.3657	3.89403	15.15	38.63	21.2400	22.8600	25.5850
SBCSII	61	452.0636	217.21438	167.67	1032.11	279.1167	384.0342	613.9493
SBCN	61	3.2223	1.00763	1.36	5.70	2.3600	3.1900	3.7800
SBCL	61	1.6546	.52378	.57	2.88	1.1950	1.5800	2.0800
SBCPLT	61	212.3770	47.14628	135.00	362.00	176.0000	211.0000	231.0000
SBCNLR	61	2.1132	.88221	.78	4.65	1.5103	1.8000	2.6115
SBCPLR	61	140.8601	54.33436	60.42	389.47	104.1653	128.2759	173.1456
