# Supplementary material for: Analysis of the diagnostic value of peripheral blood immune inflammatory indicators of female bladder pain syndrome
Source: Front Surg. 2025 Oct 31;12:1685098. doi: 10.3389/fsurg.2025.1685098 (PMC12615403; doi:10.3389/fsurg.2025.1685098)
Supplement: Supplementary file 1 [file Supplementaryfile1.zip › Appendices Table/Appendices Table2.pdf]

Eq.(2)

Tests of Normality

	Kolmogorov-Smirnov			Shapiro-Wilk		
	Statistic	df	Sig.	Statistic	df	Sig.
FBPSY	.099	149	.001	.941	149	.000
FBPSBMI	.075	149	.042	.959	149	.000
FBPSSII	.102	149	.001	.874	149	.000
FBPSN	.059	149	.200*	.982	149	.049
FBPSL	.082	149	.017	.973	149	.005
FBPSPLT	.086	149	.009	.980	149	.027
FBPSNLR	.083	149	.013	.921	149	.000
FBPSPLR	.096	149	.002	.884	149	.000
CY	.064	149	.200*	.977	149	.013
CBMI	.069	149	.078	.976	149	.011
CSII	.072	149	.056	.954	149	.000
CN	.057	149	.200*	.948	149	.000
CL	.105	149	.000	.974	149	.006
CPLT	.046	149	.200*	.965	149	.001
CNLR	.077	149	.032	.974	149	.006
CPLR	.141	149	.000	.916	149	.000

$P<0.05$

FBPS Female bladder pain syndrome Patients ;C controls Patients ;Y year ;BMI Body MassIndex;SII Systemic Immune Inflammation index ;NLR Neutrophil-to-Lymphocyte ratio;PLR Platelet-to-Lymphocyte ratio;Nneutrophil count;L absolute lymphocyte count;PLT peripheral blood platelet count
