# Supplementary material for: Analysis of the diagnostic value of peripheral blood immune inflammatory indicators of female bladder pain syndrome
Source: Front Surg. 2025 Oct 31;12:1685098. doi: 10.3389/fsurg.2025.1685098 (PMC12615403; doi:10.3389/fsurg.2025.1685098)
Supplement: Supplementary file 1 [file Supplementaryfile1.zip › Appendices Table/Appendices Table3.pdf]

Eq. (3)

DESCRIPTIVES

	Quantity	Mean	SE.	Min.	Max.	Percentile		
						25%	50%	75%
FBPSY	149	60.6913	9.57467	31.00	80.00	56.0000	63.0000	67.0000
FBPSBMI	149	23.8129	3.20771	15.15	38.63	21.6550	23.4400	26.0400
FBPSSII	149	530.7134	269.31617	156.11	2079.31	332.8100	501.1600	643.4980
FBPSN	149	3.4704	1.02743	1.34	6.60	2.7400	3.3900	4.0850
FBPSL	149	1.6623	.52620	.57	3.61	1.2850	1.5900	2.0050
FBPSPLT	149	232.5705	55.28798	94.00	400.00	190.0000	222.0000	270.0000
FBPSNLR	149	2.2524	.88345	.78	5.32	1.5600	2.1200	2.6514
FBPSPLR	149	153.8067	63.44495	60.42	480.00	109.5947	143.1600	181.4210
CY	149	59.5034	10.24604	37.00	88.00	53.5000	60.0000	66.0000
CBMI	149	24.2809	3.79090	17.60	34.66	21.3323	23.9420	26.5136
CSII	149	399.3430	166.55656	125.38	980.28	269.3634	379.6652	491.0333
CN	149	3.2185	.96538	1.48	7.95	2.4850	3.2000	3.7750
CL	149	2.1132	.49728	1.01	3.49	1.7800	2.0000	2.4650
CPLT	149	250.1007	56.54091	131.00	489.00	210.0000	245.0000	284.0000
CNLR	149	1.5717	.48049	.54	3.37	1.2308	1.5122	1.9002
CPLR	149	123.7917	37.76252	59.82	285.15	98.9609	116.8421	141.3769

FBPS Female bladder pain syndrome Patients ;C controls Patients ;Y year ;BMI Body MassIndex;SII Systemic Immune Inflammation index ;NLR Neutrophil-to-Lymphocyte ratio;PLR Platelet-to-Lymphocyte ratio;Nneutrophil count;L absolute lymphocyte count;PLT peripheral blood platelet count
