# Supplementary material for: Analysis of the diagnostic value of peripheral blood immune inflammatory indicators of female bladder pain syndrome
Source: Front Surg. 2025 Oct 31;12:1685098. doi: 10.3389/fsurg.2025.1685098 (PMC12615403; doi:10.3389/fsurg.2025.1685098)
Supplement: Supplementary file 1 [file Supplementaryfile1.zip › Appendices Table/Appendices Table4.pdf]

Eq.(4)

**Tests of Normality**

| | Kolmogorov-Smirnov | | | Shapiro-Wilk | | |
|---|---|---|---|---|---|---|
| | Statistic | df | Sig. | Statistic | df | Sig. |
| NBCY | .124 | 58 | .027 | .924 | 58 | .001 |
| NBCBMI | .120 | 58 | .037 | .949 | 58 | .017 |
| NBCSII | .158 | 58 | .001 | .808 | 58 | .000 |
| NBCN | .094 | 58 | .200* | .961 | 58 | .061 |
| NBCL | .104 | 58 | .183 | .935 | 58 | .004 |
| NBCPLT | .082 | 58 | .200* | .971 | 58 | .185 |
| NBCNLR | .137 | 58 | .009 | .871 | 58 | .000 |
| NBCPLR | .080 | 58 | .200* | .957 | 58 | .037 |
| SBCY | .135 | 61 | .007 | .931 | 61 | .002 |
| SBCBMI | .097 | 61 | .200* | .946 | 61 | .010 |
| SBCSII | .147 | 61 | .002 | .928 | 61 | .001 |
| SBCN | .103 | 61 | .177 | .976 | 61 | .286 |
| SBCL | .110 | 61 | .064 | .976 | 61 | .286 |
| SBCPLT | .089 | 61 | .200* | .973 | 61 | .193 |
| SBCNLR | .125 | 61 | .018 | .941 | 61 | .005 |
| SBCPLR | .077 | 61 | .200* | .961 | 61 | .049 |

P<0.05

*NBC normal bladder capacity ;SBC small bladder capacity ;Y year ;BMIBody MassIndex;SII Systemic Immune Inflammation index ;NLR Neutrophil-to-Lymphocyte ratio;PLR Platelet-to-Lymphocyte ratio;Nneutrophil count;L absolute lymphocyte count;PLT peripheral blood platelet count*
