# Supplementary material for: Analysis of the diagnostic value of peripheral blood immune inflammatory indicators of female bladder pain syndrome
Source: Front Surg. 2025 Oct 31;12:1685098. doi: 10.3389/fsurg.2025.1685098 (PMC12615403; doi:10.3389/fsurg.2025.1685098)
Supplement: Supplementary file 1 [file Supplementaryfile1.zip › Appendices Table/Appendices Table5.pdf]

Eq. (5)

DESCRIPTIVES

	Quantity	Mean	SE	Min	Max	Percentile 25%	50%	75%
NBCY	58	60.5000	10.85349	32.00	77.00	54.7500	63.0000	68.5000
NBCBMI	58	23.7334	2.64906	19.10	33.73	21.6925	23.4300	25.6850
NBCSII	58	606.0305	308.86183	222.68	2079.31	416.1300	556.1250	699.8775
NBCN	58	3.5647	1.05869	1.34	6.40	2.8450	3.3600	4.1350
NBCL	58	1.6581	.60209	.57	3.61	1.1675	1.5700	1.9825
NBCPLT	58	255.5690	51.12321	167.00	400.00	215.0000	248.5000	283.5000
NBCNLR	58	2.3451	.94201	.97	5.32	1.7450	2.1250	2.7996
NBCPLR	58	173.9146	69.43679	61.56	389.47	121.3575	164.5850	214.9900
SBCY	61	61.7705	8.79468	31.00	80.00	57.0000	64.0000	67.0000
SBCBMI	61	23.3984	3.89328	15.15	38.63	21.2400	22.8600	25.1800
SBCSII	61	442.0789	213.90524	156.11	1020.43	270.6050	382.0000	622.8500
SBCN	61	3.3007	1.04760	1.36	5.70	2.3600	3.2500	4.0900
SBCL	61	1.6957	.50232	.78	2.88	1.3450	1.6100	2.0800
SBCPLT	61	209.3607	46.35840	94.00	328.00	176.0000	211.0000	235.5000
SBCNLR	61	2.0872	.84201	.78	4.64	1.5103	1.9740	2.6302
SBCPLR	61	131.9852	40.88586	60.42	254.46	96.2822	125.2174	160.3061

NBC normal bladder capacity ;SBC small bladder capacity ;Y year ;BMIBody MassIndex;SII Systemic Immune Inflammation index ;NLR Neutrophil-to-Lymphocyte ratio;PLR Platelet-to-Lymphocyte ratio;Nneutrophil count;L absolute lymphocyte count;PLT peripheral blood platelet count
